# Computational repurposing of oncology drugs through off‐target drug binding interactions from pharmacological databases

**DOI:** 10.1002/ctm2.1657

**Published:** 2024-04-17

**Authors:** Imogen R. Walpole, Farzana Y Zaman, Peinan Zhao, Vikki M. Marshall, Frank P. Lin, David M. Thomas, Mark Shackleton, Albert A. Antolin, Malaka Ameratunga

**Affiliations:** ^1^ Department of Medical Oncology The Alfred Hospital Melbourne Australia; ^2^ School of Translational Medicine Monash University Melbourne Australia; ^3^ NHMRC Clinical Trials Centre University of Sydney Sydney Australia; ^4^ Garvan Institute of Medical Research St Vincent's Clinical School Faculty of Medicine UNSW Darlinghurst Australia; ^5^ ProCURE, Catalan Institute of Oncology (ICO) Oncobell, Bellvitge Institute for Biomedical Research (IDIBELL) Barcelona Spain; ^6^ The Division of Cancer Therapeutics Center for Cancer Drug Discovery The Institute of Cancer Research London UK

**Keywords:** drug repurposing, precision oncology, sequencing

## Abstract

**Purpose:**

Systematic repurposing of approved medicines for another indication may accelerate drug development in oncology. We present a strategy combining biomarker testing with drug repurposing to identify new treatments for patients with advanced cancer.

**Methods:**

Tumours were sequenced with the Illumina TruSight Oncology 500 (TSO‐500) platform or the FoundationOne CDx panel. Mutations were screened by two medical oncologists and pathogenic mutations were categorised referencing literature. Variants of unknown significance were classified as potentially pathogenic using plausible mechanisms and computational prediction of pathogenicity. Gain of function (GOF) mutations were evaluated through repurposing databases Probe Miner (PM), Broad Institute Drug Repurposing Hub (Broad Institute DRH) and TOPOGRAPH. GOF mutations were repurposing events if identified in PM, not indexed in TOPOGRAPH and excluding mutations with a known Food and Drug Administration (FDA)‐approved biomarker. The computational repurposing approach was validated by evaluating its ability to identify FDA‐approved biomarkers. The total repurposable genome was identified by evaluating all possible gene‐FDA drug‐approved combinations in the PM dataset.

**Results:**

The computational repurposing approach was accurate at identifying FDA therapies with known biomarkers (94%). Using next‐generation sequencing molecular reports (*n* = 94), a meaningful percentage of patients (14%) could have an off‐label therapeutic identified. The frequency of theoretical drug repurposing events in The Cancer Genome Atlas pan‐cancer dataset was 73% of the samples in the cohort.

**Conclusion:**

A computational drug repurposing approach may assist in identifying novel repurposing events in cancer patients with no access to standard therapies. Further validation is needed to confirm a precision oncology approach using drug repurposing.

## Graphical abstract

- Repurposing identified Food and Drug Administration‐approved drug‐biomarker combinations with high sensitivity and specificity.
- In a real‐world dataset, repurposing identified novel drug‐biomarker combinations in patients who were ineligible for standard therapies or biomarker‐matched trials.
- Preliminary functional validation was demonstrated for two drug‐biomarker combinations.
- Using The Cancer Genome Atlas data, the potential scope of repurposing was identified.

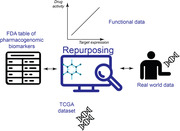

## 1 INTRODUCTION

Improving access to novel therapeutics for patients with advanced and poor prognosis cancer is challenged by logistical and efficacy impediments. Traditionally, these patients enrolled in phase I clinical trials after exhausting standard therapies. Logistically, this pathway is limited by: the application of strict inclusion criteria excluding up to two‐thirds of real‐world patients^1^ and unavailable local options in up to 77% of patients.^2^ This is compounded by limited efficacy, with aggregate response rates of 12% in early‐phase trials.^3^

To overcome efficacy limitations, biomarker enrichment leads to consistently higher response rates than agnostic approaches,^4^ consistent with the objective of precision oncology—to identify targetable molecular aberrations unique to specific patients.^5^ Biomarker enriched studies are increasingly used, with ‘umbrella’ (single condition, multiple sub‐studies for different molecular aberrations) and ‘basket’ (multiple conditions, single targetable molecular aberrations) approaches gaining prominence to improve matching patients to clinical trials.^6^ Nevertheless, these approaches are still often inapplicable to real‐world patients, for example, the National Cancer Institute – Molecular Analysis for Therapy Choice (NCI‐MATCH) umbrella study, identified ‘actionable’ alterations in 38% of patients screened, but only assigned 18% of patients to a study drug following the application of exclusion criteria.^7^ Therefore, although biomarker enrichment increases the likelihood of response to targeted therapy, the current clinical trial architecture is not permissive for access to targeted therapies in most patients.

Drug repurposing, defined as identifying new cancer indications for existing approved drugs, is an attractive alternative. Three broad types of drug repurposing have been described: off‐label use for the same molecular aberration in a different indication (such as the repurposing of trastuzumab from human epidermal growth factor receptor 2 (HER2) amplified breast cancer to HER2 amplified gastric cancer); off‐target activity (such as the use of imatinib to target *KIT* mutations in gastrointestinal stromal tumours); and combination approaches based on in vitro assays.^8^

Repurposing, if successful, has financial and logistical advantages compared to traditional drug development.^9^ Repurposing has been successful for non‐cancer indications, such as sildenafil, which was developed for angina and has been licensed for the treatment of erectile dysfunction.^10^ Few licensed repurposed therapies for oncology indications exist, with thalidomide in myeloma being the best‐described example.^9^

Most repurposing efforts have emerged from promising preclinical studies or by serendipity.^9^, ^11^ To our knowledge, systematic approaches to drug repurposing have been limited to pre‐clinical settings, such as in vitro systematic drug repurposing screens for target identification.^11^, ^12^

To our knowledge, a systematic evaluation of off‐target activity of FDA‐approved drugs to target molecular variants without approved therapies has not been undertaken. We hypothesise that systematically evaluating off‐target drug repurposing activity is feasible and potentially expands therapeutic options available to patients.

## 2 METHODS

**FIGURE 1:**
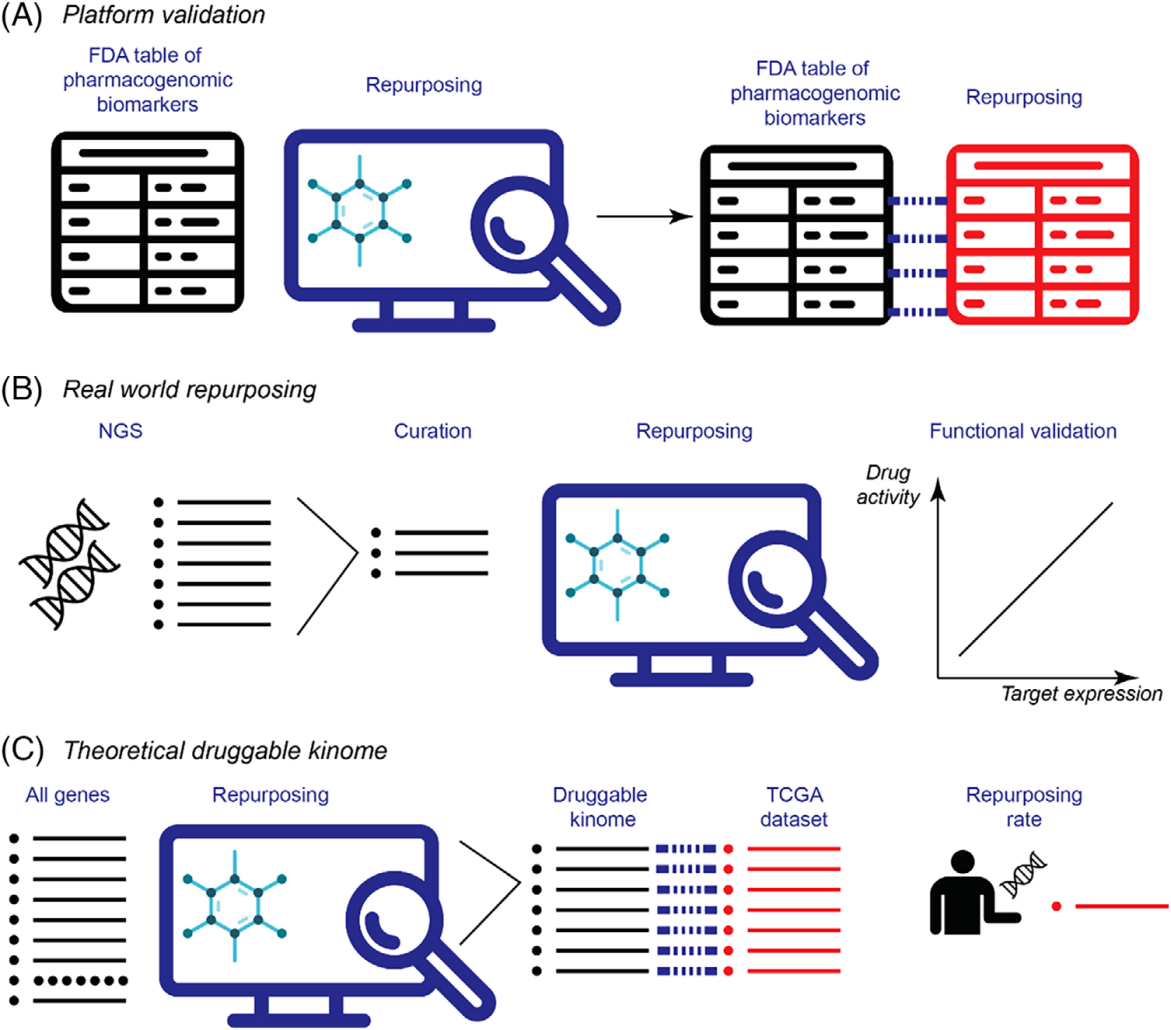
Graphical abstract of methods. (A) The accuracy of Probe Miner (PM) for identifying the Food and Drug Administration (FDA)‐approved drugs was performed by cross‐referencing a curated drug‐target combination from the FDA table of pharmacogenomic biomarkers in drug labelling and the PM database. The sensitivity, specificity and precision of PM were determined using the FDA table as the gold‐standard. (B) Real‐world patients’ next‐generation sequencing (NGS) reports were curated for gain‐of‐function mutations, with no standard therapies, and repurposing platforms were applied to identify repurposing events. Functional validation of repurposing events was performed with publicly accessible datasets. (C) The theoretical druggable kinome was determined by applying the repurposing platform to all genes for which biomarker‐selected therapies are not currently available. The frequency of aberrations in these genes in the Cancer Genome Atlas Program (TCGA) dataset was determined to calculate a theoretical repurposing rate.

A graphical abstract of the methods is shown in Figure 1. FDA‐approved oncology drugs were curated from the Table of Pharmacogenomic Biomarkers in Drug Labelling.^13^ This table was manually curated (Farzana Y. Zaman and Malaka Ameratunga) to ensure the accuracy of drug‐target combinations. For example, FDA labels relating to a tumour type (such as oestrogen receptor, *ESR1*, in breast cancer) were amended to the molecular target (such as CDK4 inhibitors). The curated table is shown in Table S1 (molecular targets labelled “Target”). This table was filtered for small molecule inhibitors (*n* = 67, Table S2).

### 2.1 Platform validation

Public databases were used to develop a systematic off‐target repurposing approach. Of note, these databases have not previously been applied to systematically evaluate off‐target repurposing in a clinical setting. We have previously developed a computational, objective, quantitative assessment of small molecules for their use to selectively study specific proteins (chemical probes): Probe Miner (PM).^14^ PM is accessible from the Institute of Cancer Research and indexes > 1.8 million compounds against 2220 human targets.^14^ PM is based on six different quantitative scores, weighted to potency and selectivity. PM uses publicly available pharmacological data from several resources integrated into the knowledgebase canSAR, which also integrates its own curated pharmacological data from selected publications.^15^ Accordingly, PM can identify the most potent and selective compound to study a specific protein (as denoted by the quantitative score 0–1). This process was undertaken to ensure that PM (via quantitative score) could correctly identify known gene‐targetable drugs (i.e. those with FDA approval).

A PM quantitative score of 0.25 for compound inclusion was used as it was slightly greater than the lower bound of the range of global scores of FDA‐approved therapies with known biomarkers. For the gold standard, curated FDA‐approved drug‐biomarker combinations were considered true positives and all non‐FDA‐approved drug‐biomarker combinations were considered true negatives to allow calculation of sensitivity and specificity. Only compounds indexed in both the Drugs@FDA database and PM were considered in this analysis as PM may not contain all the latest FDA‐approved drugs (may take months to appear in public pharmacological databases). Only biomarkers with more than one approved therapy were considered to have sufficient data to inform the analysis.

### 2.2 Real‐world repurposing

Patients with metastatic or advanced solid organ malignancies were referred from participating partner hospitals via the Monash Partners Comprehensive Cancer Consortium. Sequencing using next‐generation sequencing (NGS) through the Molecular Screening and Therapeutics (MoST) clinical trial platform study during patient care was accessed, which typically recruited patients with less common tumours. Ethics approval for the current study was granted through the Alfred Hospital Human Research Ethics Committee (HREC) (419/21).

The MoST trial NGS panel utilises either the TruSight Oncology 500 (TSO‐500) panel or the FoundationOne CDx panel. The TSO‐500 analyses 523 genes for single nucleotide variations (SNVs) and insertion/deletions (5% variant allele frequency cut‐off). It also analyses 55 genes for fusion transcripts and splice variants and can call amplifications at a limit of detection of 2.2x fold change. Alternatively, the FoundationOne CDx panel analyses 324 genes for substitutions and insertion/deletions (5% variant allele frequency cut‐off). For the FoundationOne CDx panel, amplifications are called at segments with ≥ 6 copies (or ≥ 7 for triploid/≥ 8 for tetraploid tumours) and homozygous deletions at 0 copies, in samples with tumour purity ≥ 20%. Amplifications in ERBB2 are called positive at segments with ≥ 5 copies for diploid tumours. Microsatellite instability (MSI) status was classified as high and low as per the manufacturer's specification of the respective panel. Tumour mutational burden (TMB), defined as a number of non‐synonymous mutations per megabase, was dichotomised at the threshold of 10mut/mb consistent with the KEYNOTE‐158 study that formed the basis of pembrolizumab approval.^16^

### 2.3 Classification of variants

NGS reports were reviewed by two oncologists (Imogen R. Walpole, Farzana Y. Zaman and Malaka Ameratunga) to classify and curate genomic variants of significance. Only activating (GOF) mutations were considered for the application of repurposing. Variants were classified as potential GOF or loss of function (LOF) mutations based on literature review and annotated as pathogenic, likely pathogenic, or likely benign. A literature review facilitated by the Catalogue of Somatic Mutations in Cancer database was first performed.^17^ The pathogenicity score of variants using the Functional Analysis Through Hidden Markov Models (FATHMM) was recorded.^18^ Pathogenic mutations with previous orthogonal functional validation were annotated as GOF or LOF based on the literature. For variants of uncertain significance (VUS), mutations were annotated as possibly pathogenic if they demonstrated a plausible mechanism with reference to the literature, pathogenic FATHMM score, and review by an oncologist (Malaka Ameratunga) and medicinal chemist (Albert A. Antolin). Generally, truncating mutations were classified as LOF and amplifications were classified as GOF. Non‐synonymous single nucleotide (nsSNV) variants were classified case‐by‐case, with reference to the literature. Kinase and hotspot mutations in known oncogenes were classified as potential GOF mutations.

### 2.4 Identification of drug targets

GOF mutations were assessed for possible mutation‐targeting compounds using– PM,^14^ the Broad Institute DRH^12^ and TOPOGRAPH^19^ for repurposing events. The PM was used as the primary repurposing database. The Broad Institute DRH is an annotated repurposing library combining publicly available clinical‐drug structures of > 4500 compounds from regulatory data and public databases with extensive manual curation, which has been used for in vitro screens and is available from the Broad Institute.^12^ The Broad Institute DRH was utilised to assess the degree of overlap in repurposing opportunities identified by PM and this database. TOPOGRAPH is a compendium of approved and experimental therapies assembled from regulatory data, public databases and literature review used to meet the clinical need for tiered assessment for actionability and linking biomarkers to clinical trials.^19^ It catalogues 2810 biomarker‐disease‐therapy triplets and is available from the Garvan Institute.^19^ Variants identified in PM and in TOPOGRAPH were assumed to have preclinical/clinical rationale as the TOPOGRAPH resource is designed for clinical trial allocation.

### 2.5 Categorisation of drug repurposing events

A *repurposing event* was classified as any gene aberration with a drug repurposing opportunity identified on one of the databases. GOF mutations in genes with well‐recognised, investigated and approved targeted therapies (Tier I mutations) were removed after review by two oncologists (Imogen R. Walpole and Malaka Ameratunga) and cross‐referencing with the Table of Pharmacogenomic Biomarkers in Drug Labelling published by the FDA.^13^ These included *KRAS*, *ERBB2*, *EGFR*, *BRAF*, *KIT*, *CDK4*, *CDK6* and *PIK3CA*. Mutations of potential clinical significance (aligning with Tier II^20^ mutations), for which an active ongoing clinical research program was investigating trial therapies with a strong preclinical/clinical rationale, were removed using TOPOGRAPH^19^ and annotated as *repurposing events with trial‐level evidence*. The remaining mutations were classified as *repurposing events without trial‐level evidence*. Manual curation of remaining mutations was performed by an oncologist (Malaka Ameratunga) and a medicinal chemist (Albert A. Antolin). Well‐known off‐label drug‐target interactions that are likely to be recognisable by experts (based on literature review and expert opinion) were annotated as *off‐label repurposing* events (Malaka Ameratunga and Albert A. Antolin) and drug‐target interactions which were not well‐known were annotated as *novel repurposing events*.

### 2.6 Exploratory functional analysis of drug‐target interactions

Although PM is designed to evaluate drug‐target binding, it is not designed to predict the functional consequences of this interaction. To evaluate whether prospective repurposing events are potentially functionally consequential, public datasets downloaded from the Cancer Dependency Map Project were explored.^21^, ^22^, ^23^, ^24^, ^25^, ^26^, ^27^, ^28^ The Cancer Dependency Map Project builds upon the original Cancer Cell Line Encyclopedia,^29^ which involved the systematic molecular profiling of 1000 cell lines and performed large‐scale functional genomics profiling via RNA‐interference and CRISPR screens to identify genetic functional dependencies. These cell lines had systematic drug sensitivity profiling performed, via the PRISM repurposing project and/or the Genomics of Drug Sensitivity Screens.^11^, ^21^, ^22^

Drug sensitivity data from the PRISM repurposing project^11^ and genomics of drug sensitivity screen^21^, ^22^ were cross‐referenced against gene expression data^23^ and CRISPR‐gene dependency data.^24^ Drug‐target combinations identified by PM, for which a possible relationship could be observed, were annotated. To evaluate the functional consequences of potential repurposing events, target gene expression^23^ and CRISPR dependency^24^ from the Cancer Dependency Map Project were plotted against drug sensitivity (log‐fold change in cell viability or area under the curve of a dose‐response curve), from either the Genomics of Drug Sensitivity Screens^30^ or the Broad PRISM project^11^ for repurposing events identified by the PM without trial level evidence.

To evaluate the validity of the above process for functional drug‐target interaction exploration for identified novel repurposing events, the method was tested using the FDA‐approved drug‐target database. The target gene expression^23^ and CRISPR dependency^24^ from the Cancer Dependency Map Project were plotted against drug sensitivity (log‐fold change in cell viability), from either the Genomics of Drug Sensitivity Screens^30^ or the Broad PRISM project^11^ for all known FDA‐approved drug‐target combinations (Table S1). An ANOVA was performed comparing the drug sensitivity with gene expression or CRISPR dependency data analysed by quartiles.

### 2.7 Evaluation of repurposable genome

The repurposable genome was defined as genes for which a drug repurposing event could be identified by PM (i.e. the genes with related data available in PM). This was assessed in an automated approach without manual curation, to illustrate the potential of the repurposing approach. First, the Uniprot accession indexed in PM was converted to a corresponding Entrez ID and gene name. All compounds indexed in PM per gene, with a PM global score > 0.25, were cross‐referenced against FDA‐approved therapeutics from the Drugs@FDA database (downloaded on 6 May 2022), to only include FDA‐approved drugs. The top‐ranked unique compounds (i.e. excluding compounds with known gene targets in the FDA database) were then collated with corresponding genes, to create the total repurposable genome. The genes included in the total repurposable genome were then evaluated for gene mutations in The Cancer Genome Atlas (TCGA) pan‐cancer analysis of whole genomes dataset^31^ from Cbioportal.^32^, ^33^ The TCGA dataset incorporates whole genome sequencing data, as opposed to a targeted panel, thereby identifying repurposing events at a greater frequency than the primary analysis. Due to the nature of the dataset, manual curation for pathogenic GOF mutations and removal of variants indexed in TOPOGRAPH were not performed. All analysis was performed in R version 4.2.2. Drug‐gene combinations with a PM global score > 0.7 were further analysed for potential functional drug‐target interactions using the above method.

## 3 RESULTS

### 3.1 Validation of probe miner accuracy for FDA‐approved medications

Of 67 drug‐target combinations (small molecule inhibitors) identified in the FDA Table of Pharmacogenomic Biomarkers in Drug Labelling, (Table S2), PM identified 94% as targets, with a quantitative score ranging from 0.19 (ivosidenib for *IDH1*) to 0.80 (ponatinib for *ABL1*) (Table S2). Thirty drug‐target combinations were ranked in the top ten chemical probes by quantitative score (45%), highlighting the performance of PM. PM includes many chemical probes that are not licensed drugs as its primary use is in chemical biology, so the performance for this alternative use is significant. Highly selective small molecule inhibitors consistently ranked higher than less selective inhibitors (e.g. osimertinib ranked higher than erlotinib for *EGFR*). Of the four drug‐target combinations missed by PM, one drug is rarely used (toremifene for *ESR1*).

Quantification of the performance of PM for detecting true drug‐target interactions was performed. PM identified FDA‐approved biomarker‐drug combinations with moderate‐to‐high sensitivity (range 0.5–1) and high specificity (range 0.99–1.00) (Table 1). The precision of PM was demonstrably lower, possibly relating to the high threshold used for true positives (i.e. if a drug‐target interaction identified by PM is clinically efficacious but not FDA‐listed, it would be a false positive).

**TABLE 1:** ProbeMiner identifies Food and Drug Administration (FDA)‐approved drug‐biomarker combinations.

Gene	True positive	False positive	True negative	False‐negative	Sensitivity	Specificity	Precision
CYP19A1	3	5	741	0	1	0.99	0.38
BCR	5	11	733	0	1	0.99	0.31
ALK	3	11	734	1	0.75	0.99	0.21
EGFR	5	9	735	0	1	0.99	0.36
BRAF	3	3	743	0	1	1.00	0.5
ROS1	3	8	738	0	1	0.99	0.27
ESR1	3	9	737	1	0.75	0.99	0.25
KIT	2	13	734	0	1	0.98	0.13
FGFR2	2	11	736	0	1	0.99	0.15
ERBB2	2	10	736	1	0.67	0.99	0.17
NTRK1	2	5	742	0	1	0.99	0.29
NTRK2	1	9	738	1	0.5	0.99	0.1
NTRK3	1	10	737	1	0.5	0.99	0.09
FLT3	2	14	733	0	1	0.98	0.13
PARP1	4	0	745	0	1	1	1
RET	1	17	731	0	1	0.98	0.06
MET	2	5	742	0	1	0.99	0.29
RARA	1	5	743	0	1	0.99	0.17

### 3.2 Patient demographics

Ninety‐four patients' NGS reports (September 2019 to May 2021) were reviewed. The median age of patients undergoing NGS was 63 years (Table 2). Hepatobiliary/pancreatic cancers were most common (23.4%), followed by gynaecological (21.3%), lower gastrointestinal (12.8%) and upper gastrointestinal cancers (7.4%) (Table 2). Other tumours included carcinoma of unknown primary, anal, peritoneal and thyroid cancers. Histology was most commonly adenocarcinoma (64.9%) followed by carcinoma (13.8%), squamous cell carcinoma (4.3%) and carcinoid (2.1%) (Table 2). Other histologies included papillary cancer, small cell carcinoma, serous and mucinous cystadenocarcinoma and glioblastoma. No patients were microsatellite instability‐high (Table 2). A high tumour mutational burden was found in 6.4% of patients (Table 2).

**TABLE 2:** Demographics of patient cohort.

**Age**	
Median age (range)—years	63 (29–86)
≥ 65 years—no. (%)	34 (36.2)

**Tumour stream—no. (%)**	**Total (n = 94)**
Upper gastrointestinal	7 (7.4)
Hepatobiliary/Pancreas	22 (23.4)
Lower gastrointestinal	12 (12.8)
Breast	3 (3.2)
Genitourinary	6 (6.4)
Lung	5 (5.3)
Gynaecological	20 (21.3)
Brain	1 (1.1)
Melanoma/Skin	1 (1.1)
Head and neck	2 (2.1)
Sarcoma	0 (0.0)
Other	15 (16.0)

**Tumour histology—no. (%)**	
Adenocarcinoma	61 (64.9)
Squamous cell carcinoma	4 (4.3)
Invasive ductal carcinoma	1 (1.1)
Sarcoma	1 (1.1)
Melanoma	1 (1.1)
Mesothelioma	1 (1.1)
Carcinoma	13 (13.8)
Carcinoid	2 (2.1)
Transitional cell carcinoma	1 (1.1)
Other	9 (9.6)

**Biomarker Analysis—no. (%)**	
TMB‐H	6 (6.4%)
MSI‐H	0 (0%)

Abbreviations: MSI‐H, microsatellite instability high; TMB‐H, tumour mutational burden high.

### 3.3 Mutations

Note that, 396 mutations were described from the 94 NGS reports, with 180 (45.4%) GOF mutations. The most common type of GOF alteration was amplification (58.3%), with genes most frequently affected including *CCND1*, *FGF3*, *FGF19*, *FGF4*, *MYC*, *ERBB2* and *EGFR* (Figure 1). nsSNVs represented 35.6% of GOF mutations (Figure 1). Genes most frequently altered were *KRAS*, *TP53*, *BRAF*, *PIK3CA* and *NRAS*. The frequency of fusions was 3.3% including *TMPRSS2*‐*ERG*, *HNRNPH1*‐*ETV4*, *ESR1*‐*PLEKHG1* and *TPM3*‐*ROS1*. Other alterations accounted for 2.7%.

### 3.4 Drug repurposing

**FIGURE 2:**
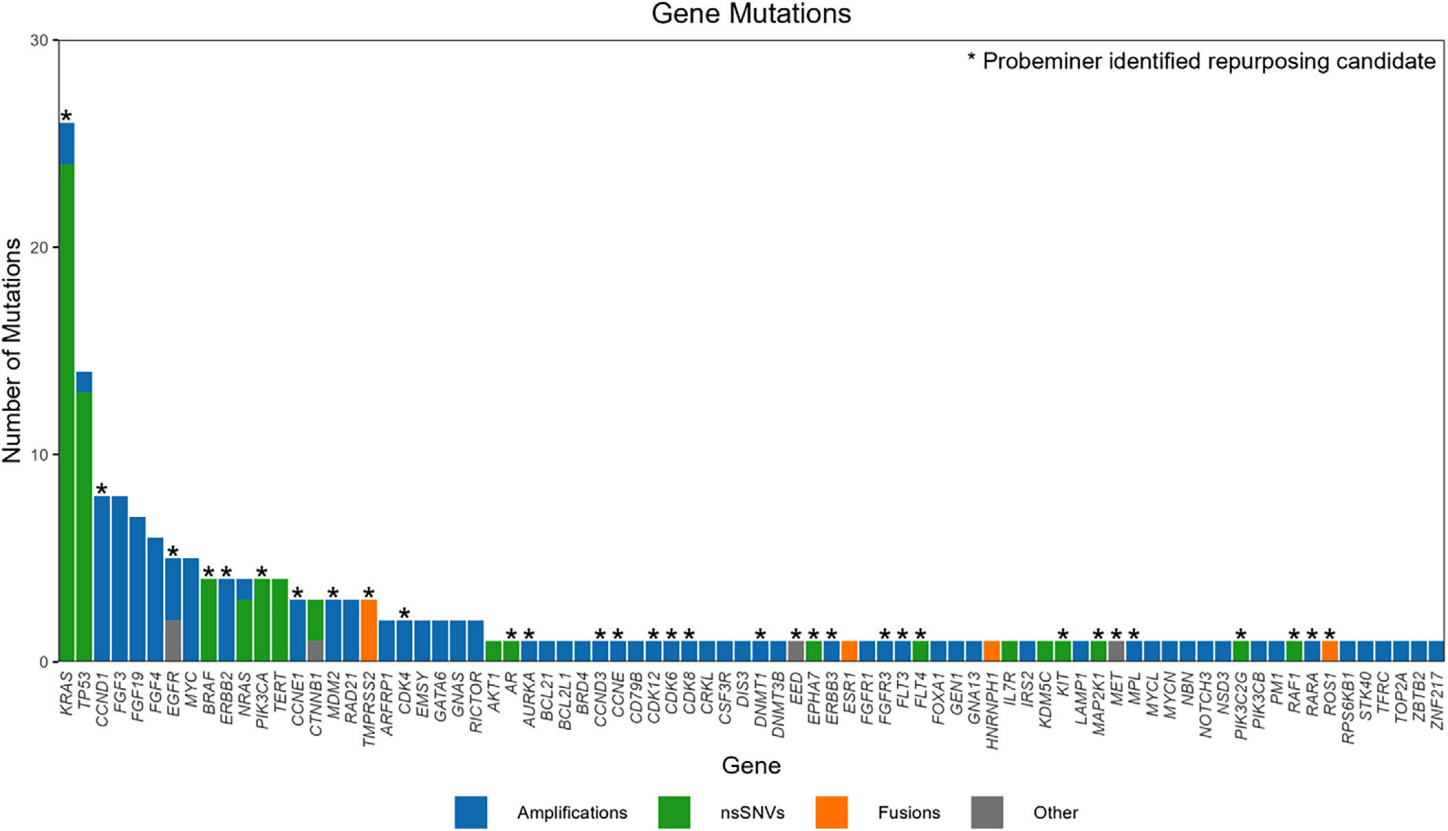
Histogram of gain of function mutations (*n* = 180). Gain of function mutations included amplifications (blue), non‐synonymous single‐nucleotide variants (green), fusions (orange) and other mutations (grey). Asterisk indicates mutations for which a repurposing event was identified on Probeminer (*n* = 32).

75 repurposing events were identified by PM, 80 by TOPOGRAPH and 50 by the Broad Institute DRH, which were reduced to 32, 29 and 26, respectively, after removing duplicates (Figure 2). Within PM, there were 21 repurposing events with trial‐level evidence, 11 repurposing events without trial‐level evidence and four novel drug repurposing events (Figure 2). At a patient level, repurposing was applied to mutations for which no FDA‐approved therapy or recruiting clinical trial was available (III). Thirteen unique patients had an off‐label gene event (i.e. the repurposing approach identified a gene target for which there was no available clinical trial nationally).

The most common types of mutations with probes found in PM were nsSNV (46.7%) and amplifications (46.7%) (Table 3, Figure 1). The most common gene involved in nsSNV mutations was *KRAS* (25.3%), with *KRAS* G12D being the most frequent. This was followed by *BRAF* (5.3%) and *PIK3CA* (5.3%) mutations. In terms of amplifications *CCND1* (10.7%), *CCNE1* (5.3%) and *ERBB2* (5.3%) were most involved. A full list of mutations identified is included in Table S3.

**TABLE 3:** Mutations with drug probes found in Probeminer.

Type of mutation	Mutated gene	Mutations (*N*)	Mutations (%)
**NSSNV**	**Total**	**35**	**46.7**
	AR	1	
	BRAF	4	5.3
	EED	1	
	EPHA7	1	
	FLT4*	1	
	KIT	1	
	KRAS	19	25.3
	MAP2K1 (MEK1)	1	
	PIK3C2G*	1	
	PIK3CA	4	5.3
	RAF1	1	
**Amplification**	**Total**	**35**	**46.7**
	AURKA*	1	
	CCND1	8	10.7
	CCND3	1	
	CCNE1	4	5.3
	CDK12	1	
	CDK4	2	
	CDK6	1	
	CDK8*	1	
	DNMT1	1	
	EGFR	5	
	ERBB2	4	5.3
	ERBB3	1	
	FGFR3	1	
	FLT3	1	
	MDM2*	1	
	MPL	1	
	RARA	1	
**Other**	**Total**	**1**	**1.3**
	MET	1	
**Fusion**	**Total**	**4**	**5.3**
	TMPRSS2‐ERG	3	
	TPM3‐ROS1	1	
	**Overall total**	**75**	

### 3.5 Novel drug repurposing events

**FIGURE 3:**
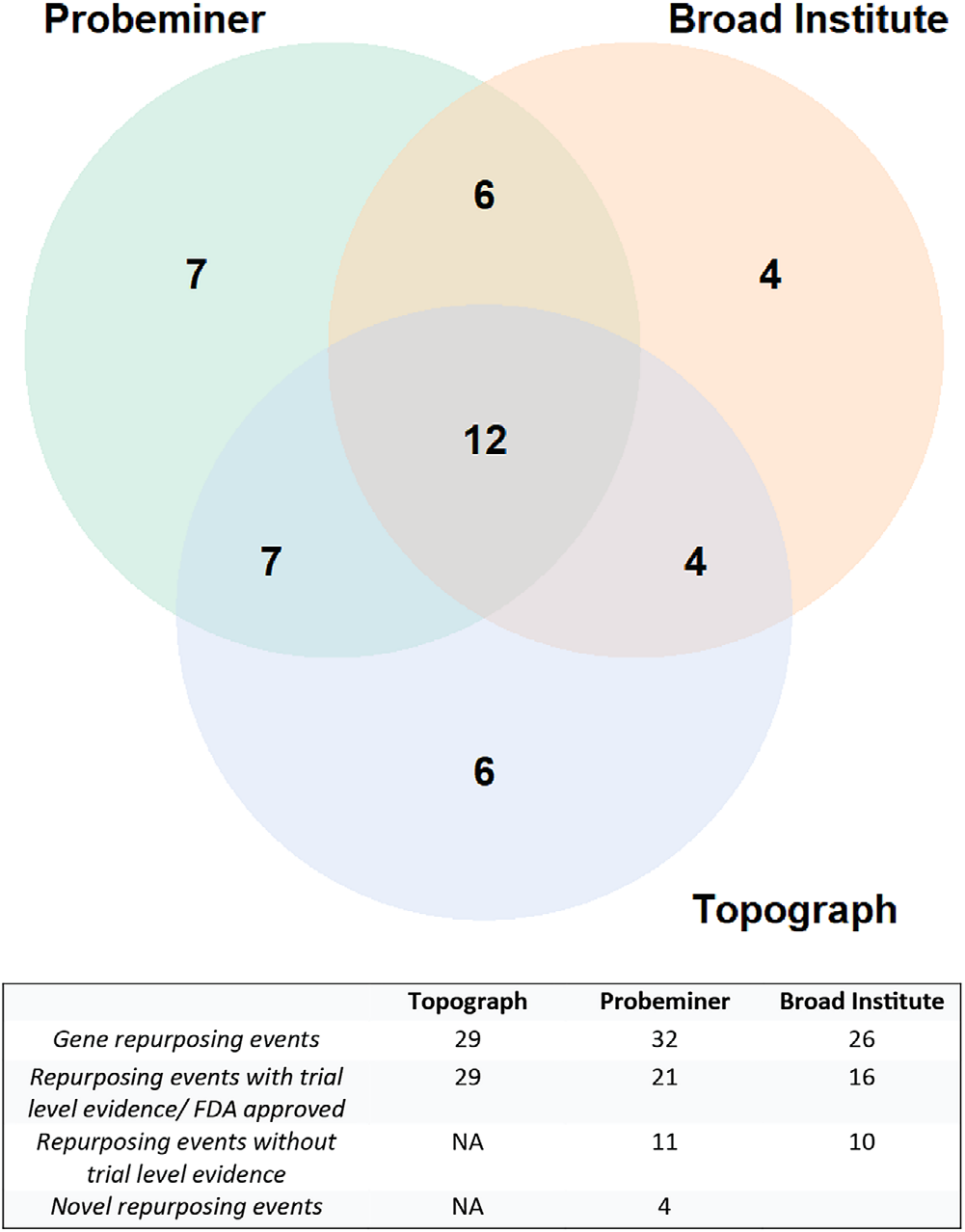
Repurposing events. Unique repurposing events identified via the three databases were evaluated for overlap as indicated in the Venn diagram (top). The breakdown of these repurposing events by trial‐level evidence and degree of novelty (bottom).

**FIGURE 4:**
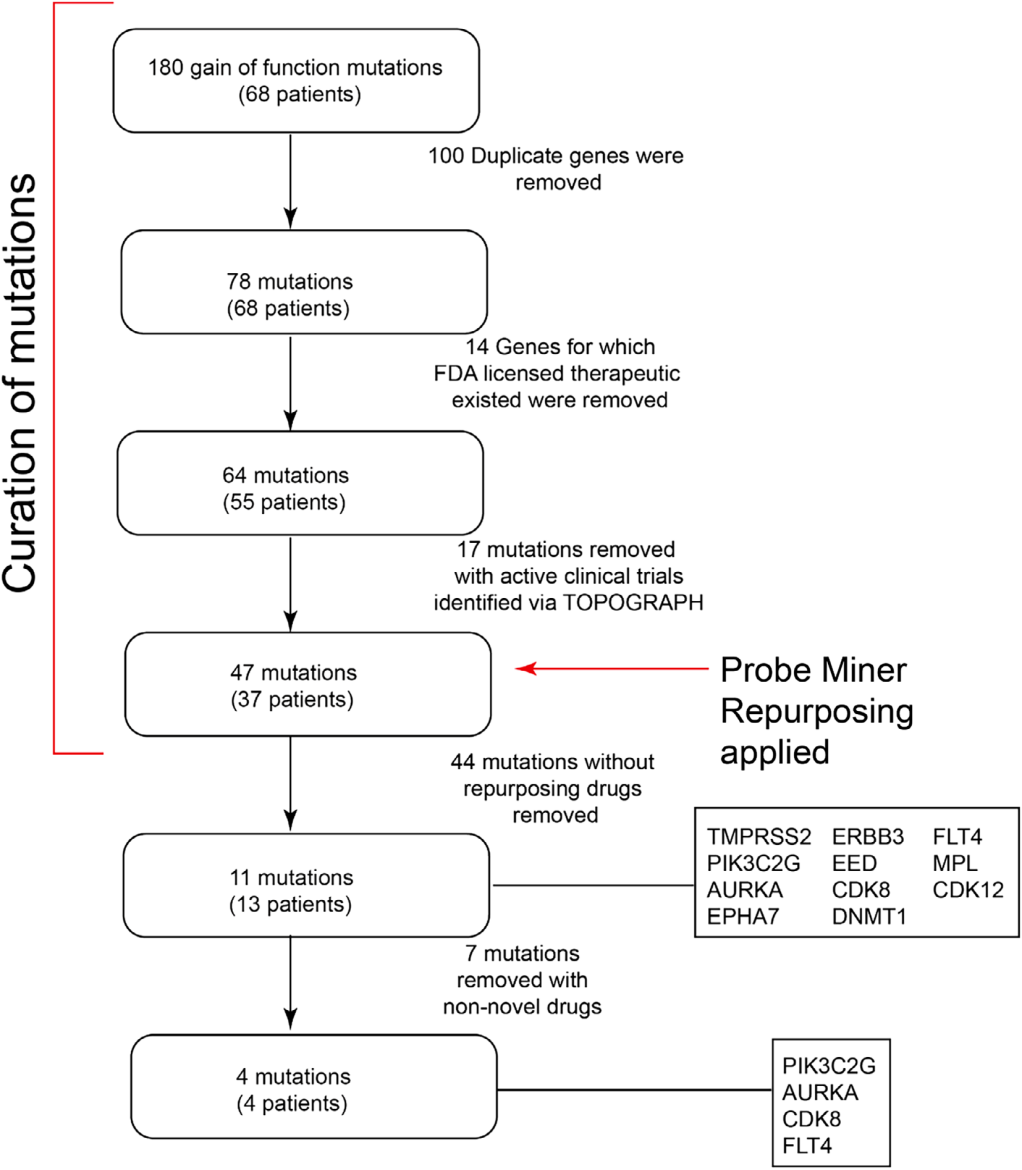
CONSORT diagram of selection of novel drug repurposing events, patient level. 180 gain of function mutations were identified across the cohort of patients, which represented 78 unique genes across 68 patients. Fourteen of these genes had the Food and Drug Administration (FDA)‐approved therapies available and a further 17 had active, locally available clinical trials evaluating this biomarker. Of the 47 remaining mutations, 11 mutations (13 patients) had a repurposable drug identified by Probeminer or which four were considered novel.

Within the PM drug events, four novel repurposing gene targets were found (Figures 3 and 4), with multiple candidate drugs, of which the top two candidate repurposing events were evaluated. The aberrations and the suggested drugs included: *PIK3C2G* R1034H (midostaurin and lapatinib) and *FLT4* V763M (axitinib and tivozanib) mutations, *AURKA* amplification (axitinib) and *CDK8* amplification (sorafenib) (Table S3). To demonstrate the validity of the drug‐repurposing events identified by PM, a comprehensive literature search was performed for the drug‐target interactions considered novel. Strong biochemical data supported drug‐target interactions in each case. For PIK3C2G‐midostaurin, PIK3C2G‐lapatinib, FLT4‐axitinib, FLT4‐tivozanib, AURKA‐axitinib and CDK8‐sorafenib experimental data demonstrating target selectivity was found.^34^, ^35^, ^36^ The potency was highest for FLT4‐tivozanib (0.2 nM) and lowest for PIK3C2G‐lapatinib (7500 nM). Overall, literature supporting each drug‐target interaction was demonstrated, with robust biochemical data observable for all novel repurposing events.

**FIGURE 5:**
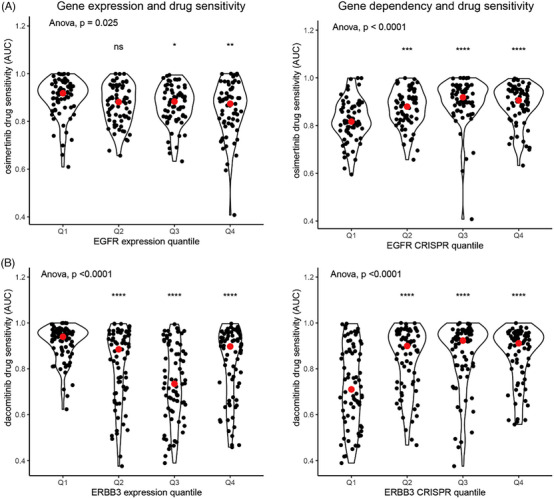
Gene expression and drug sensitivity. (A) Drug sensitivity for osimertinib (area under the curve of a dose‐response curve) according to EGFR expression (left) and EGFR gene dependency (right). (B) Drug sensitivity for dacomitinib (area under the curve of a dose‐response curve) according to ERBB3 expression (left) and ERBB3 gene‐dependency (right). Asterisks indicate the results of t‐tests comparing the relevant quartile with the first quartile of gene expression/gene dependency: * *p* < .05, **p < .01, ***p < .001, ****p < .0001.

Of the FDA‐approved drug‐target combinations, 55 had available drug sensitivity data, of which 23 (42%) had a statistically significant relationship between gene expression (*n* = 12) or gene dependency (*n* = 19) and drug sensitivity and 34 did not. Table 4 lists the FDA drug‐target combinations with statistically significant relationships between expression or CRISPR gene‐dependency data and drug sensitivity and Figure 5A (top panels) demonstrates violin plots of the drug sensitivity data for osimertinib. Only eight drug‐target combinations demonstrated a statistically significant relationship between both gene expression and CRISPR gene‐dependency and drug sensitivity, all of which were drugs targeting EGFR or ERBB2, two of the best‐characterised oncogenes. Table 4 lists all FDA‐approved drug‐target combinations for which no statistically significant relationship was found between gene‐expression/CRISPR gene‐dependency data and drug sensitivity.

**TABLE 4:** Food and Drug Administration (FDA)‐approved drug‐target combinations with supportive functional data from the Cancer Dependency Map Project.

*Genes with novel drug repurposing probes found Target/Drug combination	Gene expression and drug sensitivity *p*‐value	CRISPR‐dependency and drug sensitivity *p*‐value
**EGFR_afatinib**	**.0025**	**.0000**
**EGFR_osimertinib**	**.0250**	**.0000**
**ERBB2_neratinib**	**.0000**	**.0000**
**ERBB2_tucatinib**	**.0000**	**.0000**
**EGFR_dacomitinib**	**.0000**	**.0000**
**EGFR_gefitinib**	**.0004**	**.0000**
**ERBB2_lapatinib**	**.0126**	**.0000**
**EGFR_erlotinib**	**.0000**	**.0000**
MAP2K1_cobimetinib	.2454	.0073
BRAF_dabrafenib	.8397	.0000
BCR_nilotinib	.6058	.0127
PARP1_talazoparib	.0000	.0914
BCR_dasatinib	.9402	.0329
FGFR2_erdafitinib	.3544	0.0138
MAP2K1_trametinib	.7233	0.0117
PIK3CA_alpelisib	.0747	.0000
RARA_tretinoin	.0310	.5605
RET_cabozantinib	.5788	.0461
ESR1_fulvestrant	.7958	.0015
PARP1_niraparib	.0000	.2605
CDK4_ribociclib	.6047	.0248
PARP1_rucaparib	.0359	.8725
BRAF_vemurafenib	.3716	.0000
Total	12	19

**Bold** indicates target/drug combinations with both supporting gene expression and CRISPR‐gene dependency data.

The 11 repurposing events identified by PM (Figure 4) without trial‐level evidence underwent functional interrogation by plotting target gene expression^23^ and CRISPR dependency^24^ from the Cancer Dependency Map Project against drug sensitivity (log‐fold change in cell viability or area under the curve of a dose‐response curve), from either the Genomics of Drug Sensitivity Screens^30^ or the Broad PRISM project.^11^ A statistically significant increased drug sensitivity was demonstrated with increased *PIK3C2G* expression and lapatinib (*p <* *.001*), with *ERBB3* expression (*p <* *.0001)* and *ERBB3* gene‐dependency *(p <* *.0001)* with multiple drugs including dacomitinib. Violin plots of the gene expression and gene dependency data for dacomitinib‐ERBB3 are shown in Figure 5B (bottom panels).

### 3.6 Theoretical drug repurposing events

The total repurposable genome was evaluated by analysing all gene targets with a PM global score > 0.25 (from the PM dataset) that were indexed as an approved therapeutic in the Drugs@FDA database. All drug‐gene combinations found in this analysis are listed in Table S4. A total of 1968 theoretical repurposing events were identified. These genes were evaluated for frequency of mutations in the TCGA pan‐cancer cohort^31^ to assess the frequency of patients having possible mutations. 2142 out of 2922 (73%) samples had a mutation in a gene that was categorised as a theoretical repurposing event. Drug‐gene combinations with a PM global score > 0.7 were further analysed to identify potentially functionally consequential repurposing events. Six repurposing events with supportive functional data were found (Table 5 and Figure S1).

**TABLE 5:** Probe Miner identified (global score > 0.7) repurposing drug‐target combinations with supportive functional data from the Cancer Dependency Map Project.

Target/Drug combination	Gene expression and drug sensitivity *p*‐value	CRISPR‐dependency and drug sensitivity *p*‐value
EPHB2_dasatinib	.0079	.1454
FRK_dasatinib	.0006	.9778
FKBP1A_sirolimus	.0295	.0133
STK24_neratinib	.0014	.1545
DDR2_nilotinib	.5003	.0010
GSK3A_abemaciclib	.0766	.0392

## 4 DISCUSSION

A biomarker‐driven precision oncology approach is used to enrich patient selection for clinical trials, however, trial access issues limit the utility of this approach for real‐world patients. A drug repurposing approach, using established drugs with known safety profiles, potentially mitigates the limitations of the current paradigm. Traditional drug repurposing relies upon serendipity and more recently, systematic drug repurposing screens for drug identification.^11^ Combining genomic biomarker testing with an in silico approach utilising pre‐screened gene‐drug interaction databases could enrich the identification of drugs for repurposing to be formally tested in clinical trials. To our knowledge, systematic repurposing based on off‐target interactions has not been applied in a clinical trial setting.

In this study, using a real‐world dataset of NGS molecular reports, after demonstrating platform validation against a gold standard of FDA‐approved drugs with biomarker indications, we showed that a meaningful (14%) percentage of patients would have an additional off‐label therapeutic identified by using computational drug repurposing. This compares favourably with the results of the NCI‐MATCH clinical trial, which found an actionable alteration rate of 38%.^37^ As our computational drug repurposing excludes mutations that would confer eligibility for local clinical trials (which is the more traditional approach), this additional off‐label therapeutic access is particularly meaningful. Only 17% of patients with actionable mutations identified on the NCI‐MATCH clinical trial enrolled on a subsequent trial.^7^ Computational drug repurposing may substantially expand the number of patients treatable with a biomarker‐enriched approach including those who are typically trial ineligible. Overall, a repurposing rate of 14% was consistent with our hypothesis that computational drug repurposing may identify novel therapeutic options for patients with no further access to standard therapies.

Several exploratory analyses were conducted which raised interesting findings that require further elucidation. Firstly, several drug‐target interactions that have been previously elucidated in the medicinal chemistry literature, but are not well known, were identified. Preliminary exploratory functional analysis using publicly available datasets suggested that further validation of these targets may be warranted. Preclinical target validation is notoriously complex^38^ and whilst these results are interesting, robust additional orthogonal validation is necessary to make further conclusions on the functional consequences of drug therapy.

As licensed therapies increase continually and only 11% of the kinome is currently characterised,^14^ successful validations of this approach could potentially meet a large unmet disease burden in oncology patients as the genome is further characterised and extensive sequencing is increasingly performed. To explore the theoretical drug repurposing genome, all genes mapped to compounds with a PM global score > 0.25 with a corresponding FDA‐approved therapeutic were evaluated for their frequency on the TCGA pan‐cancer dataset. Seventy‐three per cent of samples in this cohort would have a theoretical drug repurposing event. This compares favourably with the actionable alteration rate of 38% in the NCI‐Match clinical trial. Therefore, the application of a computational repurposing approach based upon systematic off‐target interactions has the potential to drastically increase the actionability of somatic molecular alterations identified on sequencing reports.

There are several limitations to this study. In the main analysis, the curation of VUS is fraught with difficulty. Extensive manual curation with a robust framework was performed to minimise the risk of mischaracterising mutations. Additionally, as PM is predominantly based on medicinal chemistry datasets that specifically assess drug‐target binding, the assumption that drug‐target binding results in meaningful anti‐tumour activity is a large leap. Mitigating this, we evaluated PM's ability to identify approved FDA therapies linked to a biomarker, demonstrating high sensitivity (0.67–1.00) and specificity (0.99–1.00). These results supported the validity of PM. Nevertheless, although strong inferences can be made about drug‐target binding from this dataset, any conclusions regarding anti‐tumour efficacy cannot be made. Moreover, these drug‐target interactions may not be tumor‐agnostic and/or valid for multi‐drug‐resistant cancers with efflux pumps. Additionally, the drug repurposing carries inherent risks including adverse events and risk of drug interactions and does not offer a solution to the development of resistance to existing therapies. Nevertheless, in silico functional predictions of the utility of known therapies offer a novel strategy for rationally screening drug candidates to further examine in confirmatory phase 2 clinical trials.

In the exploratory analysis, robust mechanistic exploration of drug‐target interactions was not performed. To make conclusions regarding possible anti‐tumour activity, ideally in vitro cell viability assays with subsequent in vivo validation would be performed. For the analysis of the theoretically repurposable drug, the cut‐off PM global score of 0.25 was chosen as this was slightly greater than the lower bound of the range of global scores of FDA‐approved therapies with known biomarkers. The PM global score is a relative score per gene target and using an absolute score of 0.25 as a cut‐off is arbitrary. Additionally, many mutations annotated in the TCGA pan‐cancer dataset are passenger alterations and do not contribute to oncogenesis; the inclusion of such genomics findings could explain the higher‐than‐expected theoretical drug repurposing rate. Nevertheless, as the genome is further characterised and new therapies are approved these numbers will only increase and a large baseline rate of theoretical drug repurposing events is fundamentally supportive of further research in this area.

## 5 CONCLUSION

We provide initial data demonstrating that in a real‐world cohort of patients sequenced with a targeted NGS panel, 14% of patients would have a possible, non‐obvious drug repurposing candidate identified using a computational drug repurposing approach. With further study, this may be able to translate to the use of already approved therapeutics off‐label for these patients without the constraints of clinical trials.

## AUTHOR CONTRIBUTIONS

All authors contributed to the intellectual information in this manuscript and contributed to the final manuscript.

An earlier manuscript version has been uploaded to BioRxiv prior to peer review and publication (https://doi.org/10.1101/2023.07.01.547311).

## CONFLICT OF INTEREST STATEMENT

The authors declare no conflict of interest.

## FUNDING INFORMATION

No financial support has been received.

## ETHICS STATEMENT

Ethics approval for the current study was granted through the Alfred Hospital Human Research Ethics Committee (HREC) (419/21). The study was performed in accordance with the ethical standards as laid down in the 1864 Declaration of Helsinki and its later amendments or comparable ethical standards.

## Supporting information

Supporting Information

## Data Availability

Deidentified data is available upon request.
